# Young people’s health and well-being during the school-to-work transition: a prospective cohort study comparing post-secondary pathways

**DOI:** 10.1186/s12889-022-14227-0

**Published:** 2022-09-26

**Authors:** Marvin Reuter, Max Herke, Matthias Richter, Katharina Diehl, Stephanie Hoffmann, Claudia R. Pischke, Nico Dragano

**Affiliations:** 1 Institute of Medical Sociology, Centre for Health and Society, Medical Faculty, Heinrich Heine University Duesseldorf, Düsseldorf, Germany; 2 Institute of Medical Sociology, Interdisciplinary Center for Health Sciences, Medical Faculty, Martin Luther University Halle-Wittenberg, Halle, Germany; 3 Department of Medical Informatics, Biometry and Epidemiology, Medical Faculty, Friedrich-Alexander-Universität Erlangen-Nürnberg (FAU), Erlangen, Germany; 4 Mannheim Institute of Public Health, Social and Preventive Medicine, Medical Faculty Mannheim, Heidelberg University, Mannheim, Germany; 5 Department of Public Health, Faculty for Social Work, Health, and Music, Brandenburg University of Technology Cottbus-Senftenberg, Cottbus, Germany

**Keywords:** School-to-work transition, Institutional context, Vocational training, Apprenticeship, University, Prevocational preparation, Unemployment, Early career, Self-rated health, Subjective well-being, Fixed-effects, National Educational Panel Study, NEPS

## Abstract

**Background:**

At the end of secondary education, young people can either start vocational training, enter university, directly transition to employment or become unemployed. Research assumes that post-secondary pathways have immediate and/or long-term impacts on health and well-being, but empirical investigations on this are scarce and restricted to few countries. Therefore, this study traced the development of health and well-being throughout the highly institutionalised school-to-work transition (STWT) in Germany.

**Methods:**

We used longitudinal data of the National Educational Panel Study (NEPS), a representative sample of 11,098 school-leavers (50.5% girls) repeatedly interviewed between 2011 and 2020. We estimated the effect of post-secondary transitions on self-rated health and subjective well-being by applying fixed-effects (FE) regression, eliminating bias resulting from time-constant confounding and self-selection into different pathways. A multiple-sample strategy was used to account for the increasing diversity of STWTs patterns. Models were controlled for age, as well as household and residential changes to minimise temporal heterogeneity.

**Results:**

Findings indicate that leaving school was good for health and well-being. Compared with participants who did not find a training position after school, direct transitions to vocational training or university were linked to higher absolute levels of health and well-being, but also to a lower relative decline over time. Furthermore, upward transitions (e.g. to programs leading to better education or from unemployment to employment) were associated with improvements in health and well-being, while downward transitions were followed by deteriorations.

**Conclusion:**

Findings suggest that school-leave is a sensitive period and that post-secondary pathways provide young people with different abilities to maintain health and well-being. Youth health interventions might benefit when setting a stronger focus on unsuccessful school-leavers.

**Supplementary Information:**

The online version contains supplementary material available at 10.1186/s12889-022-14227-0.

## Background

The school-to-work transition (STWT) is an integral stage in life where educational pathways and early labour market experiences fundamentally determine future occupational careers [1], working conditions [2], as well as health and well-being in adulthood [3, 4]. In addition to these lifelong consequences, immediate implications of the STWT for the health and well-being of young people are possible. For instance, during the STWT, individuals are exposed to increased demands, such as finishing compulsory schooling, finding a vocational or academic training position and finally transitioning to the labour market [5]. Furthermore, young people are increasingly exposed to varying influences on health and well-being, including physical and psychosocial job demands, academic pressure, increased concerns regarding the future, and the establishment of potentially unhealthy behaviours [6, 7]. However, research focussing on the development of health and well-being throughout this critical period in life is sparse and, in particular, the influence of post-secondary pathways (e.g. the impact of the transition to vocational training, university, unemployment, or the labour market) at this life stage is understudied [8, 9]. Therefore, this article provides a longitudinal description of the development of health and well-being throughout the STWT and analyses the impact of transitions between educational institutions and labour market states on immediate changes and long-term trajectories of health and well-being. The results of this study can help identify groups of adolescents with particular health problems during the transition to adulthood, which is important for designing targeted health intervention programmes for this population.

The STWT usually covers the time between the ages of 14 to 24 years, where adolescents complete compulsory full-time education in secondary schools, move to vocational training or tertiary education, and finally transition to the labour market [5]. However, in case people do not find a training position, the transition out of school can also be followed by spells of unemployment or episodes of unskilled labour. According to assumptions made by life course epidemiology, early (labour market) disadvantage is likely to produce further disadvantage through processes of risk accumulation [10]. For instance, early unemployment was found to be a risk factor for further unemployment and poor job opportunities [11]. Those early-career “scarring effects” were debated to translate into trajectories of poor health and well-being, as labour market disadvantage and health problems are likely to reinforce each other [12]. One mechanism is that unemployment is generally associated with loss of income and social status, which often cause poverty-induced problems, such as social isolation, a loss of self-esteem, and the establishment of unhealthy behaviours [13]. Consequently, unemployment was found to increase the risk for several health problems, especially psychological disorders or respiratory and cardiovascular diseases [14]. Because good health is a necessary condition for employment, the chance for re-employment decreases with increasing duration of unemployment.

Paralleling this life course perspective, entering different institutions during STWT might also expose to different contextual influences on health [15]. Past studies show that attending higher educational tracks imparts competencies leading to better health literacy [16] and exposes to networks and social environments that are more health-promoting [17, 18]. Consequently, studies find that university students compared with trainees show more favourable health behaviours [19, 20]. In contrast, lower education often leads to employment careers involving manual labour, low income, higher physical and psychosocial job demands and elevated risks for unemployment [21–23]. Lower education is also related to lower social prestige [24] and self-esteem [25]. On the other hand, studying is often linked to academic pressure, exam stress, and prolonged financial dependence, which was found to make university students more susceptible for mental health problems [26, 27].

Despite the importance of the STWT, investigations of the development of health and well-being according to pathways entered after school-leave remain the exception. A study based on 687 Finnish adolescents reports higher well-being for school-leavers transitioning to academic compared with vocational tracks [28]. Two studies based on the US National Longitudinal Survey of Youth (NLSY97) suggest that academic study impacts positively on self-rated health [15] and body weight trajectories [29]. An analysis of the Household, Income and Labour Dynamics in Australia (HILDA) showed that transitions to unemployment after school-leave led to more disadvantaged well-being trajectories, but did not observe any differences between vocational or academic tracks [12]. One explanation for this inconsistency might stem from the heterogeneity in the institutional organisation of the STWT that is likely to produce country-specific differences [1]. Furthermore, past studies did not account for the complexity of the STWT, which is increasingly shaped by disrupted and discontinuous patterns (e.g. second-chance schooling, between-states of unemployment or unskilled labour, studying after vocational training or vice versa) [5].

This paper will address named research gaps by examining the way in which the STWT relates to health and well-being of young people in Germany. We rely on representative data of the National Educational Panel Study (NEPS) that follows 11,098 school-leavers over nine survey waves during the years 2011 to 2020. Germany provides a suitable context for studying implications of the STWT due to the availability of numerous pathways from school to work that are highly institutionalised [5]. In Germany, post-secondary education in universities is complemented with vocational education and training (VET) programs, which combine practical training in companies with theoretical education in schools [30]. Additionally, prevocational programs are available for less successful school-leavers that are unable to find a training position [31].

This study has two research objectives. The first aim is to investigate how self-rated health and subjective well-being change when people move between different STWT states (e.g. from school to vocational training or tertiary education). An advantage over previous studies is that we not merely focus on changes from school to post-school states, but also include other possible transitions (e.g. from vocational training or tertiary education to the labour market). More generally, we are interested in whether health and well-being are affected by transitions between different institutional contexts (schools, prevocational programs, vocational training places and universities) and labour market states (employment, unemployment). We assume that transitions of upward mobility (i.e. transitions to states leading to better education, e.g. from vocational training to university) relate to improvements in health and well-being, because upward transitions mark positive influences on health behaviours, employment conditions, material conditions, and psychosocial resources (e.g. self-esteem). In addition, downward transitions (e.g. to unemployment) and the associated loss of status and income are expected to negatively impact on health and well-being.

The second objective is to test for long-term consequences of different types of STWTs. Based on core assumptions of life course epidemiology [10], the transition out of school can be conceptualised as a critical period, where post-secondary pathways set the fundament for subsequent health influences, including health behaviours, labour market positions, and income opportunities. Following the assumption of risk accumulation, we expect adverse starting points after school (defined by transitions from school to unemployment or to prevocational programs) to cause more unfavourable long-term trajectories of health and well-being. In contrast, smooth and regular STWTs, defined as transitions to vocational training or tertiary education in the first year after school-leave, are expected to cause better trajectories of health and well-being.

This study uses longitudinal data in combination with a modern approach of causal inference statistics to handle several methodological challenges when studying links between educational processes and health. First, to estimate how a certain STWT state impacts on immediate and long-term changes in health and well-being, we apply fixed-effects (FE) regression and FE impact functions. As FE models only rely on changes within the same person (intra-individual variation), estimating the causal effect of a life event is possible under weaker assumptions. First, FE regression estimates are generally not biased by time-constant confounding factors, which are observed or unobserved characteristics that differ between groups of individuals and correlate with the outcome variable (i.e. time-constant heterogeneity) [32, 33]. Importantly, this approach allows for handling the problem of self-selection, resulting from the fact that educational pathways are strongly determined by characteristics such as sex, migration background, socio-economic origin, or intelligence. In particular, children of highly educated parents have a greater chance of attaining higher schooling and to enter tertiary education [34, 35]. Second, FE regression in combination with a large number of repeated measurements is more robust against bias resulting from reversed causality, which is when initial health influences educational pathways (i.e. health selection, e.g. healthier people have a higher likelihood of becoming better educated) [15]. Third, FE modelling is less affected by endogenous selection, which is when panel attrition is selective in terms of health or well-being [36]. Despite these methodological strengths of the FE approach, control must be made for time-varying heterogeneity (i.e. factors that change over time). An advantage over previous studies is that we control for possible parallel events that are interconnected with the transition to adulthood [5]. These are the general process of ageing, changes in the household composition (reflecting family ties, partnership and parenthood), and residential area changes (reflecting moving and going abroad).

Taken together, we aim to address the following two research questions:

1. How do self-rated health and subjective well-being change when moving between different STWT states?
2. How do states entered after school-leave relate to long-term trajectories of self-rated health and subjective well-being?

## Methods

### Data

We used data from Starting Cohort 4 (SC4, SUF 12.0.0) of the NEPS [37, 38]. NEPS SC4 is a representative sample of German 9th graders first interviewed in 2010 or 2011 and then followed yearly. NEPS SC4 used a stratified multi-stage sampling technique, in order to consider that the target population of 9th graders is clustered within different educational institutions [39]. A stratified sample of secondary schools was selected according to the six most common school types in Germany. Subsequently, classes were sampled within schools and then all students within those classes. Pupils were interviewed in school classes using paper-and-pencil interviews (PAPI) and school leavers were surveyed using computer-assisted telephone interviews (CATI). More detailed information on the study design and sampling procedure can be found in the study report [40]. We included all available waves up to the year 2020. We could not include the first survey wave of 2010, because self-rated health was not measured. In sum, nine survey waves between 2011 and 2020 were used, with each wave covering one calendar year (except for 2018, where no survey took place).

### Study sample

The initial sample included 92,039 person-years of 16,183 pupils. We excluded 1,137 individuals attending special needs schools, because self-rated health was not assessed in this group. Individuals were eligible for study sample when they were at least 14 years old, took part in NEPS calendar interviews, had no missing values in variables of interest, were still in school during the first person-year and were observed to leave school during the follow-up (the latter excluded participants who did not participate in the study long enough and dropped out prematurely). Eventually, 75,358 person-years of 11,098 individuals were used for the following analyses. A detailed overview of the eligibility criteria and their effect on the sample size can be found in additional file 1 (e-Table 1).

### Variables

#### Self-rated health

Self-rated health was ascertained by the question “How would you describe your health overall?” followed by a five-point Likert scale with the responses from “very poor” to “very good”. We treated self-rated health as a quasi-metric, where higher values indicate better health. Self-rated health is a global health measure reflecting overall health functioning, prevalent diseases, and current pain while predicting future mortality [41, 42].

#### Subjective well-being

Subjective well-being was measured by an adaption of the Personal Wellbeing Index for School Children (PWI-SC) [43], consisting of five 11-point scale items asking participants how satisfied they are with (i) life as a whole, (ii) standard of living, (iii) health, (iv) family, and (v) acquaintances and friends. We calculated a mean score over all five indicators ranging from 0 to 10, where higher values indicate better well-being. Subjective well-being is a proxy for mental health problems [44].

#### School-to-work transition state

After leaving the general school system, adolescents participated in biographical interviews to collect comprehensive life course data about post-secondary pathways. In each follow-up interview, participants were asked about the start and end date of each episode of education, training, or employment they had pursued. This information was stored in a specific spell format, where each data row contained one STWT episode (e.g. vocational training) in combination with the exact start and end date of the episode. We used the technique of “episode splitting” to rearrange data from spell format (which allows for several parallel states) to sequence format (where only one state per month is possible) [45]. Therefore, a priority rule was defined according to which states of vocational training and tertiary education were more important than other states. Based on the possible pathways provided by the German education system and in orientation of previous studies [31, 46], we distinguished between seven mutually exclusive STWT states: (1) school, (2), prevocational program, (3) vocational training, (4) university, (5) employment, (6) unemployment, (7) inactive (military service, civil service, parental leave). A more detailed overview of the states and the criteria applied for definitions (e.g. which training programs were defined as “vocational training”) can be found in additional file 1 (e-Table 2). Once rearrangement of biographical interview data was completed, we enriched the main data set (where each row represents a person-year) with information about the STWT states stored in the sequence data set (where each row represents a person and each column represents a month in his or her life from 14–24 years and the STWT state reached in this month) on the basis of participants’ age in months. This procedure led to a categorical, time-dependent variable that formed the basis for analysing transitional events and to identify the STWT state reached in each person-year.

#### Control variables

As mentioned in the background chapter, multiple social events are linked to the transition to adulthood, including family events and residential changes. As we are interested in the health effect of STWT states, we aim to hold other social transitions constant that might occur at the same time [5]. Thus, we control for age dummies (one life year increments), changes in the household composition and residential area changes. Age dummies were used to control for period or aging effects (e.g. controlling for a general age-related change in health and well-being over time). Information on household size and household members were used to distinguish between living with (step) parents, single-person households, couples without children, couples with children, single parents, and other households (living with other relatives or non-relatives). In case people lived with both a partner or children and parents, we coded these cases as “living with parents”. For residential change, only broad categories were available due to data protection policies (West Germany, East Germany, abroad). Note that in FE regression, observed and unobserved time-constant characteristics as sex, migration background, or socio-economic origin are automatically controlled for.

### Statistical analysis

First, we described characteristics of the study sample by presenting distributions of the dependent, independent and control variables in each survey wave through frequencies or means and standard deviations (SD) in Table 1.

**Table 1:** Sample characteristics by survey year

	**2011**	**2012**	**2013**	**2014**	**2015**	**2016**	**2017**	**2019**	**2020**
**Observations**									
Individuals (n)	10,334	10,042	10,158	9,545	9,091	8,206	7,408	5,844	4,730
**Gender**^**a**^									
Male (%)	49.5	50.0	49.8	49.5	49.8	49.3	48.7	49.3	48.6
Female (%)	50.5	50.0	50.2	50.5	50.2	50.7	51.3	50.7	51.4
**Age (years)**									
Mean	15.1	15.9	16.7	17.5	18.7	19.7	20.6	22.6	23.6
(SD)	(0.6)	(0.7)	(0.7)	(0.7)	(0.7)	(0.7)	(0.7)	(0.7)	(0.6)
**Self-rated health**									
Mean	4.1	4.1	4.2	4.2	4.2	4.2	4.2	4.2	4.1
(SD)	(0.9)	(0.8)	(0.8)	(0.8)	(0.8)	(0.8)	(0.7)	(0.8)	(0.8)
**Subjective well-being**									
Mean	8.1	8.0	8.3	8.3	8.3	8.3	8.4	8.2	8.2
(SD)	(1.6)	(1.5)	(1.2)	(1.1)	(1.0)	(0.9)	(0.9)	(0.9)	(0.9)
**STWT state**									
School (%)	100.0	88.3	60.1	57.3	24.1	5.9	2.3	0.7	0.4
Prevocational program (%)	0.0	4.5	7.3	3.7	1.8	1.0	0.7	0.2	0.2
Vocational training (%)	0.0	6.4	28.0	32.0	38.0	33.1	29.0	14.4	10.1
University (%)	0.0	0.0	0.0	0.1	14.5	34.1	41.0	45.8	47.1
Employment (%)	0.0	0.4	1.7	3.2	12.3	19.0	22.4	35.2	38.9
Unemployment (%)	0.0	0.2	1.4	2.3	3.6	3.5	2.8	2.6	2.4
Inactive (%)	0.0	0.2	1.5	1.4	5.7	3.5	1.8	1.0	0.9
**Region**									
West Germany (%)	87.8	87.5	88.2	88.3	87.3	82.9	81.2	79.1	78.3
East Germany (%)	12.2	12.5	11.8	11.7	11.9	16.0	17.3	19.0	19.2
Abroad (%)	0.0	0.0	0.0	0.0	0.8	1.1	1.4	1.9	2.6
**Household**									
Living with parents (%)	94.6	95.7	96.8	95.7	85.2	73.9	64.4	45.1	36.7
Single-person household (%)	0.0	0.1	0.9	1.5	7.0	12.3	15.9	21.5	26.6
Couples without children (%)	0.0	0.1	0.5	1.0	3.0	5.2	8.7	18.6	23.8
Couples with children (%)	0.0	0.0	0.0	0.0	0.0	0.0	0.0	0.1	0.2
Single parents (%)	0.0	0.0	0.0	0.1	0.2	0.3	0.4	0.4	0.4
Other households (%)	5.4	4.2	1.6	1.6	4.5	8.2	10.6	14.4	12.3

Data set: NEPS SC4, SUF 12.0.0. *n* = 11,098 individuals with 71,358 person-years. Number of individuals (n), column percentages (%) or means and standard deviations (SD)

^a^ Time-constant variable

For the purpose of answering research questions, we applied linear fixed-effects (FE) regression analysis for panel data [32, 33]. FE regression relies only on intra-individual variation over time and allows investigating how an outcome changes if a person changes from a control (e.g. school) to a treatment group (e.g. university). By using only within-variation, FE regression is not biased by between-individual heterogeneity that is constant over time. Thus, we control in our analyses for multiple characteristics that are associated with STWT state and health and could otherwise confound effect estimates (e.g. sex, migration background, parental education, personality, intelligence, characteristics of teachers, classes or schools). Furthermore, as we allow for multiple person-years in each state, the estimation of person-specific intercepts is more robust against health-related selection (reversed causality) [15]. Finally, FE regression estimates are even unbiased in case of endogenous selection bias, which is present in case of panel attrition patterns associated with the outcome variable (e.g. higher likelihood for early dropout in case of poor health or well-being) [36]. A Hausman test further supported to choose a FE model over a model with random effects (χ^2^ = 343.02, df = 25, *p* < 0.001).

The analytical strategy contained two steps. For the first research question, that is to test if health and well-being are affected by transitions between different STWT states, we estimated regression models for each outcome with STWT state as a multi-categorical time-varying predictor. The state before a transition occurred was defined as the reference category. Taking into account the possibility of multiple transitional events, a single estimation strategy with school as the only reference state would not allow to study other transitions that are possible. A solution for this problem is to split the data set into multiple samples and to analyse the effect of each transition using only person-years that store information on this specific transition. We used six subsamples (S1-S6) capturing each of the six states of main interest (school, prevocational program, vocational training, university, employment, and unemployment) in combination with the person-years of the state entered afterwards. We allowed for multiple person-years in the same state to minimise reverse causality bias. An exemplary data set for two participants is shown in the additional file 1 (e-Table 3).

**Fig. 1:**
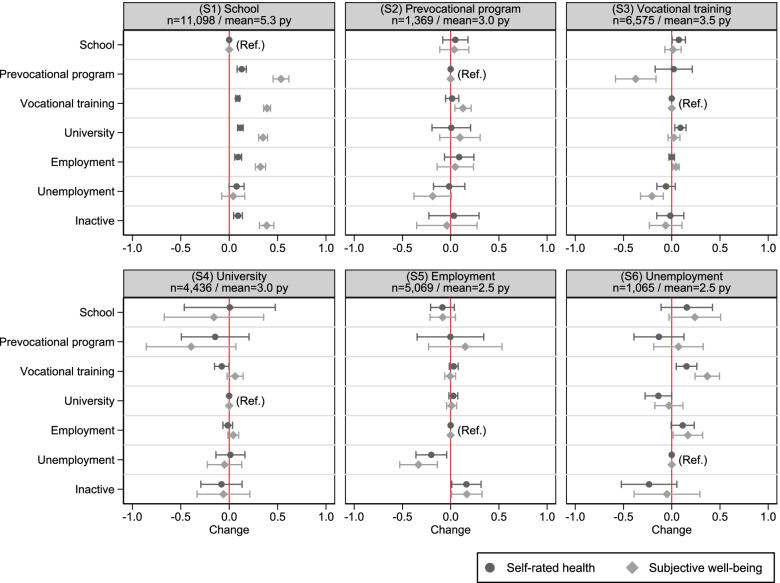
Changes between STWT states and their impact on self-rated health and subjective well-being. Data set: NEPS SC4, SUF 12.0.0. Results of Table 2. Regression coefficients and 95% confidence intervals of six linear fixed-effect analyses with cluster-robust standard errors. *n* = number of individuals. Py = person-years. Time-varying controls: Age dummies, region of education or work, and household composition. The effect of transitional events on health and well-being were investigated in different estimation samples (S1-S6) that include the person-years of the reference state and the person-years of the state that was entered afterwards

Models were controlled for age (dummies with one life-year increments), and area and household changes to reduce time-varying heterogeneity between individuals (parallel trends or exogeneity assumption) [33]. To correct for serial autocorrelation and heteroscedasticity, we specified all FE models with cluster-robust standard errors. Results are shown in Table 2. In order to facilitate the interpretation of multiple regression estimates, we also plotted results as average marginal effects (AMEs) [47] in Fig. 1.

**Table 2:** Linear fixed-effects regression analysis for self-rated health and subjective well-being

	**Self-rated health**						**Subjective well-being**					
	**S1** **b/(SE)**	**S2** **b/(SE)**	**S3** **b/(SE)**	**S4** **b/(SE)**	**S5** **b/(SE)**	**S6** **b/(SE)**	**S1** **b/(SE)**	**S2** **b/(SE)**	**S3** **b/(SE)**	**S4** **b/(SE)**	**S5** **b/(SE)**	**S6** **b/(SE)**
**STWT state**												
School	Ref	0.05	0.07*	0.01	-0.09	0.16	Ref	0.04	0.01	-0.16	-0.08	0.24
		(0.07)	(0.04)	(0.24)	(0.06)	(0.14)		(0.08)	(0.04)	(0.26)	(0.07)	(0.14)
Prevocational program	0.13***	Ref	0.02	-0.14	0.00	-0.13	0.53***	Ref	-0.37***	-0.39	0.15	0.07
	(0.02)		(0.10)	(0.18)	(0.18)	(0.13)	(0.04)		(0.11)	(0.24)	(0.19)	(0.13)
Vocational training	0.09***	0.02	Ref	-0.08*	0.03	0.15**	0.39***	0.13**	Ref	0.06	0.00	0.37***
	(0.01)	(0.03)		(0.04)	(0.02)	(0.05)	(0.02)	(0.04)		(0.04)	(0.03)	(0.06)
University	0.12***	0.01	0.09**	Ref	0.03	-0.14*	0.35***	0.10	0.02	Ref	0.01	-0.03
	(0.01)	(0.10)	(0.03)		(0.02)	(0.07)	(0.02)	(0.11)	(0.03)		(0.03)	(0.07)
Employment	0.09***	0.09	0.00	-0.02	Ref	0.11	0.32***	0.05	0.04**	0.04	Ref	0.17*
	(0.02)	(0.08)	(0.01)	(0.03)		(0.06)	(0.03)	(0.10)	(0.02)	(0.03)		(0.08)
Unemployment	0.08	-0.02	-0.06	0.01	-0.20*	Ref	0.04	-0.19	-0.20***	-0.05	-0.33***	Ref
	(0.04)	(0.08)	(0.05)	(0.08)	(0.08)		(0.06)	(0.10)	(0.06)	(0.09)	(0.10)	
Inactive	0.09***	0.03	-0.01	-0.08	0.16*	-0.23	0.39***	-0.04	-0.06	-0.06	0.17*	-0.05
	(0.02)	(0.13)	(0.07)	(0.11)	(0.08)	(0.15)	(0.04)	(0.16)	(0.09)	(0.14)	(0.08)	(0.17)
**Region**												
West Germany	Ref	Ref	Ref	Ref	Ref	Ref	Ref	Ref	Ref	Ref	Ref	Ref
East Germany	-0.01	-0.29	-0.02	0.05	0.01	0.08	-0.02	-0.08	0.06	0.05	0.05	-0.08
	(0.02)	(0.16)	(0.04)	(0.03)	(0.03)	(0.10)	(0.03)	(0.15)	(0.04)	(0.03)	(0.04)	(0.11)
Abroad	-0.05	0.47*	0.00	0.05	0.12	0.22*	-0.01	-0.12	0.01	-0.04	0.17**	0.00
	(0.05)	(0.24)	(0.11)	(0.05)	(0.07)	(0.11)	(0.06)	(0.36)	(0.11)	(0.05)	(0.06)	(0.20)
**Household**												
Living with parents	Ref	Ref	Ref	Ref	Ref	Ref	Ref	Ref	Ref	Ref	Ref	Ref
Single-person household	-0.02	0.20*	0.01	-0.01	-0.03	0.02	0.06*	0.09	-0.07**	0.01	-0.10***	0.00
	(0.02)	(0.09)	(0.02)	(0.02)	(0.03)	(0.07)	(0.02)	(0.09)	(0.03)	(0.02)	(0.03)	(0.09)
Couples without children	0.01	0.14	0.01	0.00	0.03	0.11	0.13***	0.08	0.08***	0.06*	0.08*	0.21*
	(0.02)	(0.08)	(0.02)	(0.03)	(0.03)	(0.08)	(0.03)	(0.10)	(0.02)	(0.03)	(0.03)	(0.11)
Couples with children	0.06	0.00	0.11	-0.96***	0.01	-0.03	0.58	0.00	0.16	-0.94***	0.32	0.40*
	(0.55)	(0.00)	(0.22)	(0.02)	(0.19)	(0.23)	(0.47)	(0.00)	(0.14)	(0.02)	(0.17)	(0.18)
Single parents	0.06	0.51	0.12	-0.11	-0.12	0.14	0.02	0.83	-0.29	0.42	0.02	-0.10
	(0.15)	(0.32)	(0.19)	(0.30)	(0.14)	(0.18)	(0.34)	(0.51)	(0.19)	(0.21)	(0.17)	(0.18)
Other	-0.02	0.11	-0.02	-0.01	-0.03	0.08	0.00	-0.20	-0.07*	0.01	-0.04	-0.07
	(0.02)	(0.08)	(0.03)	(0.02)	(0.03)	(0.08)	(0.03)	(0.17)	(0.03)	(0.02)	(0.04)	(0.11)
**Intercept**	4.09***	4.16***	4.15***	4.21***	4.16***	3.84***	8.01***	8.46***	8.38***	8.36***	8.38***	7.86***
**Model information**												
R-squared (within)	0.007	0.016	0.005	0.005	0.009	0.029	0.038	0.030	0.024	0.025	0.022	0.039
Individuals (n)	11,098	1,369	6,575	4,436	5,069	1,065	11,098	1,369	6,575	4,436	5,069	1,065
Person-years (n)	58,542	4,138	23,284	13,467	12,531	2,676	58,542	4,138	23,284	13,467	12,531	2,676

Data set: NEPS SC4, SUF 12.0.0. b = Regression coefficient (positive values indicate increases). *SE* Standard error. *Ref* Reference category. The effect of transitional events on health and well-being were investigated in different estimation samples (S1-S6) that include the person-years of the reference state and the person-years of the state that was entered afterwards. Each model includes age dummies as controls, with the median age in each subsample as the reference category (not shown)

^*^ *p* < 0.05

^**^ *p* < 0.01

^***^ *p* < 0.001

**Fig. 2:**
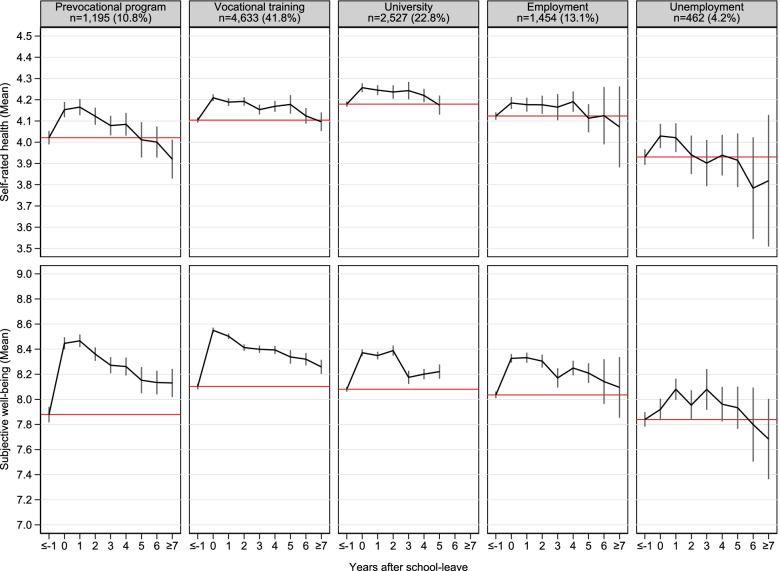
Trajectories of self-rated health and subjective well-being by state reached after school-leave. Data set: NEPS SC4, SUF 12.0.0. Adjusted predictions at the mean (APMs) and 95% confidence intervals of ten linear fixed-effect regressions with cluster-robust standard errors. *n* = number of individuals. Time-varying controls: Region and household composition. Red horizontal line represents predicted averages of health and well-being during school. To test how different states entered after school-leave affected trajectories of health and well-being, fixed-effects impact functions were estimated stratified by state reached in the year “0”. For transitions to “inactivity”, no subplot is shown. For the sample of university students, no estimate was calculable for year 6 or higher due to low case number

For the second research question, that is to analyse trajectories of health and well-being in dependence of the state entered after school-leave, we used FE impact functions [48]. The main predictor was an event-centred time scale, which was derived by subtracting the interview date in each person-year with the date of the school-leave (value “0” indicates the first year out of school). Separate impact functions were calculated by state reached in the year “0” and subsequently converted into adjusted predictions at the means (APMs) [47] visualised in Fig. 2. A plot showing the proportion of states in each year after school-leave is to find in additional file 1 (e-Fig. 1).

All analyses were performed using Stata 16.1 MP (64-bit, StataCorp LLC, College Station, TX, USA).

## Results

### Sample description

Table 1 provides an overview of the characteristics of study participants in each survey wave. Over time, the number of participants declined from 10,334 to 4,730, while the mean age increased from 15.1 to 23.6 years. Over the study period, participants transitioned from school to different post-school states. By the end of the survey period, most of participants were either in university (47.1%), employed (38.9%), in vocational training (10.1%), or unemployed (2.4%). As also indicated by this table, the share of participants living with parents decreased over time and was at 36.7% by the end. Furthermore, health and well-being increased over time and finally decreased by the end of the study period.

### Impact of STWT states on health and well-being

Table 2 shows the results of the FE regression analysis for self-rated health and subjective well-being. It is apparent from the analysis of the first sample (S1) that leaving school was associated with a significant improvement in health and well-being. This increase was observable for participants who transition to a prevocational or vocational training program, to university, directly to employment or to inactivity. In contrast, no change was observed when transitioning to unemployment. In addition, attendees of prevocational and vocational training programs experienced a stronger increase in well-being compared with university students or those directly entering work. If we now turn to the regression coefficients concerned with the transition to employment (S2, S3, S4), self-rated health appeared to be unaffected when starting a job after (pre)vocational training or university. However, a slight positive effect on subjective well-being was found when entering work after a vocational training program.

Now moving to Fig. 1 that visualises estimates of Table 2, we can observe that moving from a vocational training program to university had a positive impact on self-rated health (S3). In contrast, a change from university to vocational training decreased self-rated health (S4). We also found that transitions from prevocational programs to vocational training had a positive effect on subjective well-being (S2). In contrast, moving from vocational training to a prevocational measure was negative for well-being (S3). Furthermore, a transition from unemployment to employment or vocational training was followed by better health and well-being (S6). In accordance, a change from employment or vocational training to unemployment (S3, S5) appeared to have negative effects. Furthermore, no significant effect on health and well-being was found when re-entering school (S2-S6).

### Trajectories of health and well-being after school-leave

Figure 2 illustrates trajectories of self-rated health and subjective well-being by state reached after school-leave. As indicated by the sample size reported in each subplot, most people transitioned directly to vocational training (41.8%), university (22.8%), or to a prevocational measure (10.8%). In addition, some participants also started working without any training (13.1%), or transitioned to unemployment (4.2%) or inactivity (7.5%, not shown). The FE impact functions modelling strategy also supports the previous result that school-leave was linked to increases in health and well-being (despite for people who transitioned to unemployment). Furthermore, this increase was rather of short duration, as a decline in health and well-being over the subsequent years was apparent. In case of transitions to vocational training or university, the decline was less steep, while people entering prevocational programs or unemployment reached their school-levels of health and well-being earlier. Accordingly, we found that participants moving from school to prevocational measures or to unemployment were more likely for subsequent spells of unemployment afterwards (e-Fig. 1). Furthermore, as indicated by the red horizontal line, participants who entered unemployment or a prevocational program had lower averages of health and well-being even before school-leave compared with vocational trainees or university students. Furthermore, university students showed better self-rated health compared with trainees.

FE impact functions stratified by gender indicate that health and well-being were higher for males compared with females, while males exhibited a steeper decline compared with females (e-Fig. 2).

## Discussion

This study was set out to investigate the intra-individual development of health and well-being over the course of the STWT in a sample of German school-leavers. The first research question sought to determine how self-rated health and subjective well-being were affected by transitions between STWT states. Overall, findings indicate that leaving school was positive for health and well-being, irrespective if participants entered a prevocational program, vocational training, university, employment or inactivity after school. Two other studies from Finland and Germany found similar results for subjective well-being [28, 49]. Accordingly, a study from Canada observed decreases in the prevalence of depression during the same time period [50]. An explanation is given with reference to an assumption of life course research, according to which transitional events can be positive for health in case they resolve an unfavourable situation [51]. In this case, ending compulsory schooling could reflect a relief of exam stress or mark the end of uncertainty in finding a training position. Consequently, increases in well-being were stronger for attendees of prevocational programs, who are generally those with the greatest uncertainty before school-leave. A second explanation is given with reference to the social production function theory [52]. Leaving compulsory education means for young people to firstly follow their own goals and preferences, and therefore to experience a gain in autonomy, status control, and behavioural confirmation positively linked to physical and mental well-being. In contrast to our findings, a study from Australia did not observe changes in subjective well-being after leaving secondary schooling [12], which could reflect variations by local structures of the education and labour market system. For instance, school pressure might be higher in countries with VET systems that produce stricter barriers for later labour market entries [5].

A further finding related to the first research question was that transitions of upward mobility (i.e. from a prevocational program to vocational training, from vocational training to university, from unemployment to employment) were positive for health and well-being, while downward transitions (i.e. from vocational training to a prevocational program, from university to vocational training, from employment to unemployment) were negative. This might be explained by the loss of status and income associated with unemployment, or the negative experience of training or university dropout [15].

The second question in this research was if states entered after school-leave affect trajectories of health and well-being. FE impact functions demonstrated that health and well-being declined over the years after school-leave. However, a smooth STWT (i.e. from school to vocational training or university) was related to a decline that was less fast compared with an unsuccessful STWT (i.e. from school to unemployment or a prevocational program). In addition, participants who were unsuccessful in finding a training position exhibited lower averages of health and well-being even before school-leave, causing trajectories that were more disadvantaged in terms of absolute levels, but also in terms of relative change over time. This finding was also obtained in the Australian HILDA study [12] and accords with cumulative risk assumptions of life course epidemiology [10]. Furthermore, we observed that trajectories of self-rated health were more favourable for university students compared with attendees or vocational training programs. This accords with other studies comparing self-rated health [15, 53] and weight trajectories [54] between vocational and academic tracks. Possible reasons are that institutions of higher and lower education differ in their socio-structural compositions [17, 18], relevant in terms of social norms and health behaviour [19, 20], but also in terms of curriculums linked to health literacy [16]. Thus, studying mediation via compositional and contextual factors bound to the institutions (i.e. behavioural, material, psychosocial factors) is an important issue for future research.

### Strengths and limitations

This study has several limitations and strengths. First, as we relied on global health measures, we are not able to identify specific somatic or mental diseases. A second limitation is the change of the survey mode for school-leavers from PAPI to CATI. Thus, the positive effect of school-leave might be underestimated in our study, as health assessments are prone for upward bias in personal interview settings through social desirability [55]. A third limitation is given with regard to the survey period, in which a larger part of the university students had not yet entered work. Thus, future research should extend the study period above the age of 24 years.

Nevertheless, this was one of the first longitudinal studies on health and well-being during the STWT using detailed information on pathways attended after school-leave. As we used panel data in combination with FE regression, we were able to account for several methodological challenges when investigating educational processes and their relationship with health, including time-constant between-individual heterogeneity [32, 33], endogenous selection [36], and reversed causality [15]. Second, this was the first study applying a multiple sample strategy, which gave new insights into a rising segment of STWTs shaped by more discontinuous transition patterns. Third, as we controlled for transitions occurring parallel to STWTs (household and residential changes), we minimised the possibility of bias by temporal heterogeneity [36].

## Conclusions

Taken together, findings of this study indicate that post-secondary pathways entered by young school-leavers seem to be highly important for their health and well-being. First, transitions to programs leading to ‘more’ education, as well as transitions out of unemployment were found to impact positive on young people’s health and well-being. Second, findings highlight the STWT as a period of high sensitivity with regard to pathways entered after school-leave and their long-term impact on health and well-being. It seems that institutions of higher education provide young people with networks and knowledge necessary to promote and maintain health. Results of this study might be helpful when developing targeted youth health intervention programs. First, findings point to higher health needs of unsuccessful school-leavers who are not able to find a training position. Second, this case study from Germany provides evidence that youth labour market interventions can also be beneficial for health promotion, as it has been found that prevocational programs cause better trajectories of health and well-being compared with direct transitions to unemployment.

## Supplementary Information

**Additional file 1: e-Table 1**. Assessment for eligibility for study sample. **e-Table 2**. Criteria used to define nine mutually exclusive states during STWT. **e-Table 3**. Example for dividing the data set into multiple samples (S1-S6) for two participants. **e-Figure 1.** Proportion of states in each year after school-leave stratified by state entered in the first year out of school. **e-Figure 2.** Trajectories of self-rated health and subjective well-being after school-leave by gender**. **Data set: NEPS SC4, SUF 12.0.0. Regression coefficients and 95% confidence intervals of linear fixed-effect analysis with cluster-robust standard errors. Time-varying controls: Region of education or work, and household composition. Red horizontal line (0) represents average health and well-being during school.

## Data Availability

The data that support the findings of this study are available from NEPS Research Data Center but restrictions apply to the availability of these data, which were used under license for the current study, and so are not publicly available. Data are however available from the authors upon reasonable request and with permission of the Leibniz Institute for Educational Trajectories (LIfBi) at the University of Bamberg (Germany).

## Abbreviations

AME: Average marginal effects
APM: Adjusted predictions at the means
CAPI: Computer-assisted personal interview
CATI: Computer-assisted telephone interviewing
FE: Fixed-effects
NEPS: National Educational Panel Study
SC4: Starting Cohort 4
SUF: Scientific use file
STWT: School-to-work transition
