# Supplementary material for: Young people’s health and well-being during the school-to-work transition: a prospective cohort study comparing post-secondary pathways
Source: BMC Public Health. 2022 Sep 26;22:1823. doi: 10.1186/s12889-022-14227-0 (PMC9511745; doi:10.1186/s12889-022-14227-0)
Supplement: Supplementary file 1 — Additional file 1: e-Table 1. Assessment for eligibility for study sample. e-Table 2. Criteria used to define nine mutually exclusive states during STWT. e-Table 3. Example for dividing the data set into multiple samples (S1-S6) for two participants. e-Figure 1. Proportion of states in each year after school-leave stratified by state entered in the first year out of school. e-Figure 2. Trajectories of self-rated health and subjective well-being after school-leave by gender. Data set: NEPS SC4, SUF 12.0.0. Regression coefficients and 95% confidence intervals of linear fixed-effect analysis with cluster-robust standard errors. Time-varying controls: Region of education or work, and household composition. Red horizontal line (0) represents average health and well-being during school. [file 12889_2022_14227_MOESM1_ESM.docx]

# Additional file 1

e-Table 1 Assessment for eligibility for study sample

|  | **Included** | | | | |  | **Excluded** | |
| --- | --- | --- | --- | --- | --- | --- | --- | --- |
|  | **Individuals** | |  | **Person-years** | |  | **Ind.** | **Prys** |
|  | **n** | **%** |  | **n** | **%** |  | **n** | **n** |
| Original sample | 16,183 | 100.0 |  | 92,039 | 100.0 |  | - | - |
| Drop pupils of special needs schools | 15,046 | 93.0 |  | 87,580 | 95.2 |  | 1,137 | 4,459 |
| Drop if missing information on variables of interest | 14,983 | 92.6 |  | 86,584 | 94.1 |  | 63 | 996 |
| Drop if age <14 years | 14,983 | 92.6 |  | 86,552 | 94.0 |  | 0 | 32 |
| Drop individuals that had left school in the first person-year | 14,488 | 89.5 |  | 84,485 | 91.8 |  | 495 | 2,067 |
| Drop individuals that were not observed to leave school* | 11,098 | 68.6 |  | 75,358 | 81.9 |  | 3,390 | 9,127 |

* Concerned participants who did not participate in the study long enough and dropped out prematurely.

e-Table 2 Criteria used to define nine mutually exclusive states during STWT

| **State** | | **Detail** |
| --- | --- | --- |
| 1 | School | Elementary school, orientation stage, *Hauptschule, Realschule, Mittelschule*, *verbundene Haupt- und Realschule* (also *Wirtschaftsschule, Regionale Schule,* extended *Realschule*, R*ealschule plus, Gemeinschaftsschule, Werkrealschule, Stadtteilschule*) , *Gymnasium*, comprehensive school (also integrated schools and dual secondary school), Waldorf school, and vocational school (in case they vitis only leads to a general school-leaving qualification, e.g. *Fachoberschule*), other school forms |
| 2 | Prevocational program | Includes forms of vocational preparation for people out of compulsory education but having problems to find vocational training positions: *Berufsvorbereitungsjahr* (BVJ), *Berufsgrundbildungsjahr* (BGJ), vocational preparation training measure (BvB), *Berufsfachschule*, short entry qualification year, career start year (BEJ) |
| 3 | Vocational training | Apprenticeship including practical training in a company or other non-school location (dual vocational training; administrative, in-company, industrial, agricultural), master craftsman/craftswoman or technician, vocational training program at school of public health, *Berufsfachschule*, or *Fachschule* |
| 4 | University | Studying at a university of applied science, administration academy, business academy, or similar, or at regular university (including technical university, medical university, theological college, teacher training college, veterinary college as well as colleges of music and art) |
| 5 | Employment | Working for pay or profit for an employer or being self-employed |
| 6 | Unemployment | Out of school, but not in employment or further education and seeking for work |
| 7 | Inactive | Military, civil service, or parental leave |

e-Table 3 Example for dividing the data set into multiple samples (S1-S6) for two participants

| **Person** | **Person- year** | **Age** | **State** | | **S1 School** | **S2 Prevocational program** | **S3 Vocational training** | **S4 University** | **S5 Employment** | **S6 Unemploy-ment** |
| --- | --- | --- | --- | --- | --- | --- | --- | --- | --- | --- |
| 1 | 1 | 15 | 1 | School | 1 (Ref.) |  |  |  |  |  |
| 1 | 2 | 16 | 1 | School | 1 (Ref.) |  |  |  |  |  |
| 1 | 3 | 17 | 2 | Prevocational program | 2 | 2 (Ref.) |  |  |  |  |
| 1 | 4 | 18 | 3 | Vocational training |  | 3 | 3 (Ref.) |  |  |  |
| 1 | 5 | 19 | 3 | Vocational training |  | 3 | 3 (Ref.) |  |  |  |
| 1 | 6 | 20 | 3 | Vocational training |  | 3 | 3 (Ref.) |  |  |  |
| 1 | 7 | 21 | 5 | Employment |  |  | 5 |  | 5 (Ref.) |  |
| 1 | 8 | 22 | 6 | Unemployment |  |  |  |  | 6 | 6 (Ref.) |
| 1 | 9 | 23 | 5 | Employment |  |  |  |  |  | 5 |
| 2 | 1 | 16 | 1 | School | 1 (Ref.) |  |  |  |  |  |
| 2 | 2 | 17 | 1 | School | 1 (Ref.) |  |  |  |  |  |
| 2 | 3 | 18 | 1 | School | 1 (Ref.) |  |  |  |  |  |
| 2 | 4 | 19 | 4 | University | 4 |  |  | 4 (Ref.) |  |  |
| 2 | 5 | 20 | 4 | University | 4 |  |  | 4 (Ref.) |  |  |
| 2 | 6 | 21 | 4 | University | 4 |  |  | 4 (Ref.) |  |  |
| 2 | 7 | 22 | 4 | University | 4 |  |  | 4 (Ref.) |  |  |
| 2 | 8 | 23 | 4 | University | 4 |  |  | 4 (Ref.) |  |  |
| 2 | 9 | 24 | 5 | Employment |  |  |  | 5 |  |  |

Ref. = reference category.


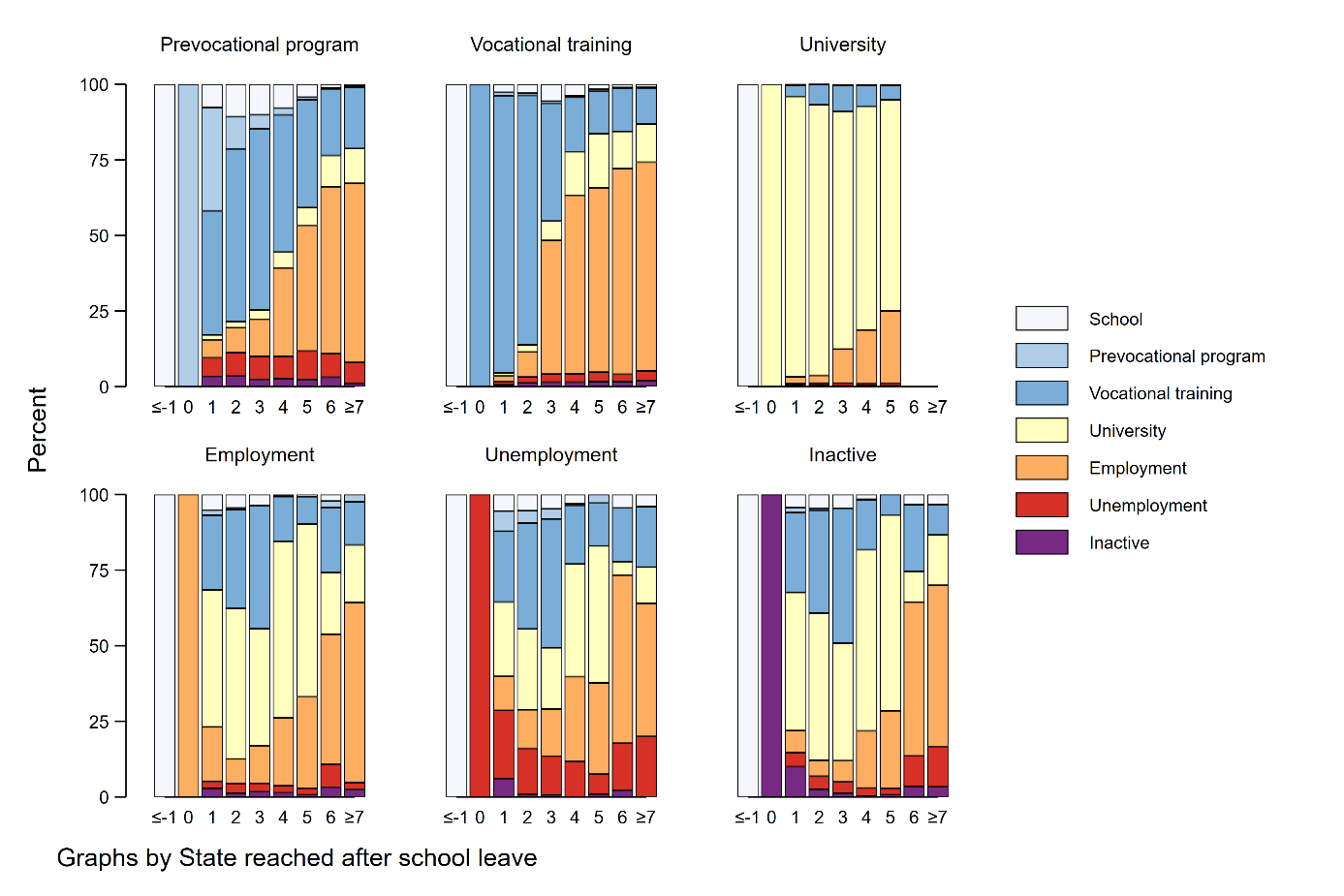


e-Figure 1 Proportion of states in each year after school-leave stratified by state entered in the first year out of school.


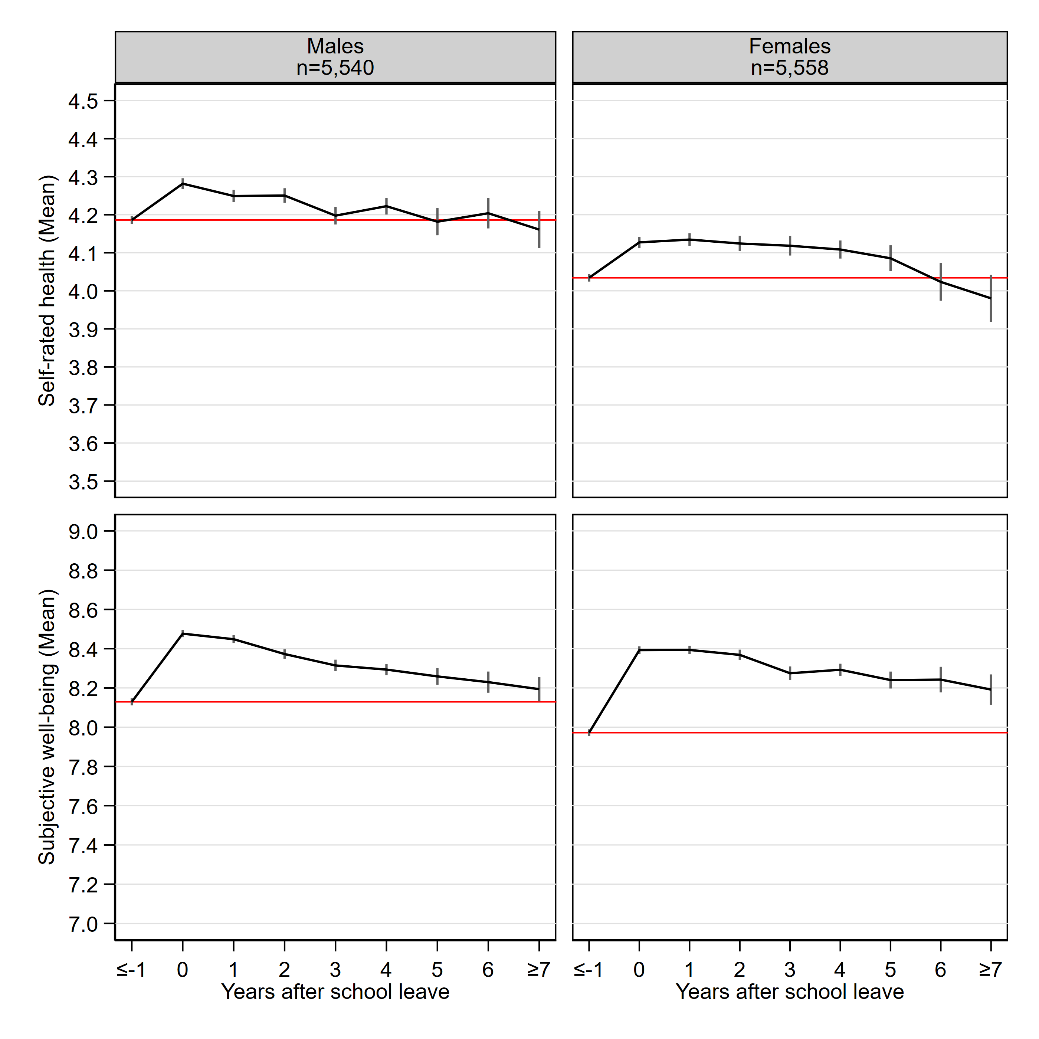


e-Figure 2 Trajectories of self-rated health and subjective well-being after school-leave by gender

Data set: NEPS SC4, SUF 12.0.0. Regression coefficients and 95% confidence intervals of linear fixed-effect analysis with cluster-robust standard errors. Time-varying controls: Region of education or work, and household composition. Red horizontal line (0) represents average health and well-being during school.
